# Can We “Brain-Train” Emotional Intelligence? A Narrative Review on the Features and Approaches Used in Ability EI Training Studies

**DOI:** 10.3389/fpsyg.2021.569749

**Published:** 2021-08-17

**Authors:** Ming D. Lim, Mabel C. Lau

**Affiliations:** ^1^School of Psychology, University of Sydney, Sydney, NSW, Australia; ^2^School of Psychology, University of Wollongong, Wollongong, NSW, Australia

**Keywords:** ability emotional intelligence, MSCEIT, cognitive training, brain training, emotional competencies, emotional processes

## Abstract

Recent studies had demonstrated that specific emotional intelligence (EI) abilities (as measured using the MSCEIT) were related to better performance on cognitive tasks that involved emotional information but not on their non-emotional counterparts. These findings suggest that cognitive control and other executive functioning processes (e.g., working memory) contribute to EI abilities. A well-functioning EI ability is crucial for a number of everyday activities and life outcomes. However, the evidence for training ability EI remains vague as to how these improvements occur. The purpose of this narrative review was to synthesize findings from past EI training research, specifically focusing on their methodology. This was to identify key aspects of the interventions used, to determine the prototypical features between them, as well as to propose a compelling research agenda for future EI training studies. Based on the features found in these studies, we identified two possible approaches in which EI improvements occurred. The first approach was through increasing emotional knowledge and related competencies through teaching and practice. These features were found in the majority of training interventions using a workshop-style training format, reflections, role-plays, and practice with other participants. The second approach used brain-training principles to improve basic cognitive processes, such as executive control or emotional inhibition. Using a cognitive training approach to EI training can provide several advantages, such as allowing researchers to examine EI improvements using the theories of (1) transfer; (2) plasticity; and (3) process-specific changes.

## Introduction

Emotional intelligence (EI) broadly refers to a set of competencies in the processing, understanding, and regulation of affective information whether toward oneself or others (Mayer et al., 1999). Recently, several experimental and cross sectional studies have demonstrated that EI abilities significantly influenced performance on cognitively “hot” tasks as compared to cognitively “cold” tasks (e.g., working memory updating for happy faces as compared to neutral-to-balanced or non-emotional stimuli, such as faces with a neutral expression, letters, or shapes) (Gutiérrez-Cobo et al., 2017a,b; Lim and Birney, 2021). Such findings are intriguing, in that they highlight that EI consists of a set of abilities that are utilized when there is a need to work with emotional stimuli. Importantly, they also suggest a way to conceptualize EI training by using an approach typically utilized by brain training researchers (e.g., working memory training).

Two research questions motivated this review: (1) What processes and abilities have been targeted in previous EI training interventions; and (2) whether there is a method to improve EI abilities using a cost-effective or ecological manner while retaining the rigor, reliability, and validity expected of psychological research. Using a narrative approach, we sought to identify the features or commonalities found in past ability EI training studies. Based on these features, we identified two broad approaches in which ability EI could be improved upon: (1) through the development of long-term emotional knowledge by increasing emotional literacy; or (2) by cognitive training of processes subserving EI abilities. We also presented several studies that contained the prototypical elements for these two approaches. This article is written primarily for researchers in the ability EI domain and for those who are interested in the cognitive training of such abilities.

In total, we identified 25 training studies that utilized an ability EI measure or used the EI model approach for its training. Of these, we identified two broad approaches (as mentioned above). In Approach 1, we found three features that were consistently used. Specifically, an educational instruction methodology (e.g., classroom teaching) using a variety of training activities was adopted. The key focus appears to be on experiential learning. Thus, these training programs tend to require a substantial duration to complete. Importantly, we identified a second approach in ability EI training, specifically process-based training using a cognitive framework. Ability EI training using this approach differs substantially in terms of methodology.

## Trait vs. Ability Emotional Intelligence

In general, there are two distinct perspectives on the conceptualization of EI: Trait or ability EI. The trait EI perspective states that EI is about one's self-perceptions and dispositions regarding their own emotional skillsets (Petrides and Furnham, 2000). The key method of classification within this perspective is the use of self-report questionnaires, where items measure typical perceptions in emotion-related situations. In our review, we focused predominantly on EI as defined by the ability perspective for two reasons. First, several excellent systematic reviews and meta-analyses of EI had already been published in recent years. Kotsou et al. (2019) completed a comprehensive systematic review of 46 EI intervention studies (consisting of only adults) and assessed their efficacy in improving various life outcomes. Likewise, Hodzic et al. (2018) identified 24 studies to determine the effectiveness of training EI and the factors that could contribute to its success.

Together with Schutte et al. (2013) original meta-analysis on EI training, these articles concluded that such interventions worked and were moderately useful for improving EI. However, the focus for these reviews had been mostly on examining how effective the interventions were (i.e., “what is the efficacy of the EI training”), but not on the mechanisms in which these improvements were built upon. Many of the past EI training studies utilized interventions that were atheoretical at best or often lacked theoretical justification on why the training had improved EI of the participants (e.g., a visit to an art gallery as part of the EI training; Dacre Pool and Qualter, 2012). This was in contrast to the broader scientific literature on cognitive training, where relatively clear paradigms and justifications existed as to why specific abilities had been targeted upon (e.g., using working memory tasks to improve performance on fluid reasoning tests, Jaeggi et al., 2009).

Second, we chose to review EI training because this perspective views EI as an intelligence that is based on or built upon by our abilities to use emotional information. From this perspective, EI consists of a class of abilities that involves several emotion-related processes (Mayer et al., 2016). O'Connor et al. (2019), in their critical review on EI measurements, suggested that the ability EI perspective is best used when researchers are interested in emotional abilities and their theoretical models. This approach has important implications for researchers interested in training EI. For a training to be deemed as effective, it is important that the purported improvements fulfill several criteria, such as being measurable on different objective criteria scales or having active control groups (Jacoby and Ahissar, 2013).

The use of objective or performance-based measures (within the ability EI framework) also allows researchers to quantify improvements or differences in training outcomes. For example, if a person were to be trained on the accuracy for judging another person's emotional states, we would also expect better performance on the Perceiving Emotion branch on the Mayer–Salovey–Caruso Emotional Intelligence Test (MSCEIT) (as reported by Herpertz et al., 2016). On the other hand, trait EI—similar to other personality traits—could be largely immutable and somewhat resistant to change despite a prolonged training duration. For instance, Sander et al. (2017) found that traits such as Openness to Experience and Conscientiousness did not improve after 100 days of daily 1-h cognitive training of memory and perceptual speed.

## Review and Synthesis Methodology

Kotsou et al. (2019) provided the most up-to-date systematic review of the effectiveness of EI training. Using the Problem, Intervention, Comparison, and Outcome (PICO) framework, they identified 46 studies that met the criteria of EI training and nine of these studies used an ability EI measure. As such, our present review followed Kotsou et al.'s search strategies and examined each of these studies for the possible training approaches used in these studies. For the purpose of the current review, we utilized techniques from the narrative review approach (Wong et al., 2013). This review process is designed to review topics that have been conceptualized differently by groups of researchers. Wong et al. (2013) suggested that this method could highlight the relative strengths and limitations of studies being reviewed. Finally, a narrative (or semi-systematic) review is useful for detecting the theoretical perspectives or commonality within a specific methodology, as well as to identify common components of a concept (Snyder, 2019).

Using PsychInfo, SCOPUS, and ScienceDirect, we searched for studies using the keywords emotional intelligence, ability emotional intelligence, MSCEIT, training, intervention, cognitive training, brain training, improvements, and outcomes. However, we removed studies that did not include an ability EI measure or did not explicitly mention the training of EI using the ability perspective. Apart from the MSCEIT, we also searched for training studies that utilized other ability EI measures, such as the Situational Test of Emotional Understanding (STEU) and the Situational Test of Emotional Management (STEM) (MacCann and Roberts, 2008). Additionally, we examined studies that are more recent because Kotsou et al. (2019) had searched for relevant training studies published until June 2016.

Following a narrative approach, we also included training studies that involved participants from diverse groups, such as adolescents, inpatients, and outpatients as these studies could also provide information regarding the approaches used for EI training. The key difference between a narrative review and a systematic review is that the latter method has pre-specified criteria for including or excluding studies (Snyder, 2019). Since we were interested in the broad features used in training ability EI, the only predetermined criteria were the exclusions of studies that did not include an ability measure.

### Screening and Analysis

Both authors individually searched each paper to first determine the methodology and the EI models used. We paid particular attention to the methodology sections, the protocols used for training, and the specific activities used during the training period. We then synthesized these findings by identifying the features found in these studies. These features were coalesced into categories or themes in order to identify the commonality between these studies. Following a thematic approach (Braun and Clarke, 2006), we did not attempt to address the efficacy of these studies (as was previously done by Kotsou et al., 2019 and Hodzic et al., 2018); rather, the focus was to identify the similarity or the prototypical elements between them. The two authors discussed all of these features until an agreed classification was reached.

## Results

The review identified the same set of studies used in Kotsou et al. (2019), including eight training studies that used the MSCEIT as a training outcome measure. An additional 14 papers were identified for a total of 25 studies (see Table 1 for the list of studies). The authors agreed on four features, with the first three identified as almost universally present in all of the ability EI studies as listed in Kotsou et al. (2019) study. As such, these were classified under Approach 1: Teaching ability EI. The features were (a) educational teaching format; (b) constellation of activities (e.g., feedback, group work, self-reflection, role-plays); and (c) duration of study completion. The fourth feature: computerized training methods^1^ was present predominantly in the studies that utilized cognitive training principles to justify their training protocols. While these studies could also require a similar duration to complete, the core training activity was almost singular in nature and format (i.e., computerized training). Thus, these studies were grouped under Approach 2: Brain-training ability EI.

**Table 1:** Overview of ability emotional intelligence training studies used in the present narrative review.

**Studies found in Kotsou et al. (2019)**	**Sample**	**Ability EI Measure**	**Control Group**	**Active Control**	**Randomization**	**Initial Training Format**	**Activities during or after the initial training**
Clarke (2010a)	MBA students	MSCEIT	Yes	No, control condition only completed 1 day workshop	No	1 day EI training workshop on Self-awareness	14 weeks of team-based activities to complete a report: Reflections and discussions
Clarke (2010b)	Project managers	MSCEIT	No	No active control group	No	2 days of EI workshop training	Not provided, though the authors mentioned the use of feedback and structured exercises to improve EI
Codier et al. (2011)	Nurse Manager	MSCEIT	No	No control group	No	Monthly informational sessions	Weekly peer sessions, reflection, discussions
Codier et al. (2013)	Nurses	MSCEIT	No	No control group	No	Sharing sessions between participants and two staff members of the research team	EI peer-sharing of 5 minutes over 10 months
Crombie et al. (2011)	Cricket Players	MSCEIT	Yes	No active control group	No	10 sessions of EI-related training using a workshop format, each session lasted 3 h	Not provided, though the authors reported that the sessions were “informative and participative,” with time for participants to share with their peers regarding the EI topic for that training
Dacre Pool and Qualter (2012)	College Students	MSCEIT	Yes	No active control group	No	11 sessions of weekly EI classes, each class lasted for about 2 h	Lectures, role plays, group discussions, and readings about EI-related topics
Meyer et al. (2004)	Dental practice employees	MSCEIT	No	No control group	No	1 day of 'experiential' EI course	Participants completed indoor and outdoor ropes and a physical “challenge course. Trained facilitators conducted “discussion sessions” during and after completion of each activity
Nelis et al. (2009)	College students	STEU	Yes	No active control group	No	Weekly classes of EI for 4 weeks, each class lasted 2.5 h	Lectures, role plays, group discussions, readings about topics, and two-person sharing. Each participant was given a personal diary to report one emotional experience for each day
Nelis et al. (2011)	College Students	STEU and ERP-R	Yes	No active control group	No	Three 6 h sessions of EI workshops or Six 3 h workshops	Similar to Nelis et al. (2009)
Peter and Brinberg (2012)	Students with weight issues	MSCEIT	Yes	Yes, using a “bogus” training workshop	Not stated	One 75 min EI-session	Not provided, but the authors followed Elias and Arnold (2006) recommendations. As such, the activities included lectures, group work, sharing, role-plays, and discussions
Wagstaff et al. (2013)	Sports professionals	STEU	No	No control group	No	3 EI workshops over 18 weeks. Each workshop lasted between 2 and 3 h	One-to-one coaching for 3 months, but for only three participants
**Additional Studies Included in the Present Review**							
	**Sample**	**Ability EI Measure**	**Control Group**	**Active Control**	**Randomization**	**Initial Training Format**	**Activities during or after the initial training**
Antoun et al. (2020)	Doctors in Residential Training	MSCEIT	No	No control group	No	20 Balint seminars in 1 year	Lectures to content specific to Balint workshops
Castillo-Gualda et al. (2017)	Teachers in two schools	MSCEIT	Yes, one school	No, waitlist control method	No	8 sessions of 3 h training using the RULER approach	Lessons, worksheets, role-plays practice, reflection
Eack et al. (2007)	Inpatient and Outpatients	MSCEIT	Yes	Yes	Yes	60 h of computerized brain training in attention, memory, and problem-solving	45 social-cognitive group sessions to learn about theory of mind, reading social cues, and emotional management
Geßler et al. (2020)	University students	MSCEIT	Yes	Active control group: Organization course	Yes	1 day workshop + 4 weeks of online activities (role-plays, reflection, practice)	4 weeks of online activities: role-plays, reflection, practice
Gilar-Corbí et al. (2019a)^3^	Higher Education students	STEU	Yes	No, as participants in the control group did not complete any training or non-training activities.	Yes	95 min of face-to-face teaching over 7 weeks, with access to an online “virtual training.”	A trained researcher provided training and education regarding 7 domains of emotional abilities (e.g., emotional perception)
Gilar-Corbí et al. (2018a)	Trainee teachers	MSCEIT	Yes	No, as control group participants continued in their usual activities	Yes	95 min per session of face-to-face training over 8 weeks	Each session, participants completed activities and learning on various emotional intelligence abilities
Gilar-Corbí et al. (2018b)	Higher Education students	STEU	Yes	No: Not clearly specified, but appears to be waitlist control group	Yes	Minimum of 1 hourly session per week over 7 weeks	The authors utilized the Emotional Intelligence Training Program” (EITP) in three possible modalities (online, in the classroom, and coaching). Participants in the classroom and coaching group gets additional hours per session each week
Gilar-Corbí et al. (2019b)	Senior managers of a private company	STEU	Yes	No, as participants in control group did not complete any training or non-training activities	Yes	95 min per session of face-to-face teaching over 7 weeks, with access to an online “virtual training.”	A trained researcher provided training on various emotional intelligence abilities
Herpertz et al. (2016)	University students	MSCEIT	Yes	Active control group: Time management course	Yes	1 day training on emotional perception	4 weeks of online activities, divided into two types: Emotional Perception training; and role-plays (using mirrors. These role-plays were recorded on video and participants reflected on their performance
Hooker et al. (2013)	Inpatient and Outpatients	MSCEIT	Yes	Yes, computer games training	Yes	60 min of Computerized Auditory Training + 5 to 15 min of computerized Social-cognition training	Participants continued with the computerized training individually daily for 10 weeks
Köppe et al. (2019)	University students	MSCEIT	Yes	No: Waitlist control group	Yes	1 day face to face training	4 weeks of practice activities online (role-plays, videos)
Martyniak and Pellitteri (2020)	Teachers in Poland	MSCEIT	Yes	No: Waitlist control group	Yes	3 monthly full-day EI workshops	Short daily exercises, involving reflections, learning about emotions, videos, role-plays
Nahum et al. (2014)	Enrolled in an early psychosis program	MSCEIT	Yes	Yes, healthy matched controls completed SocialVille program	Yes	60 min of daily computerized training exercises using the SocialVille program for ~20 h of training	Participants continued with the computerized training individually daily, 2 to 5 time each week for 6 to 12 weeks
Pozo-Rico and Sandoval (2020)	Trainee teachers	Not measured	Yes	No, control group participants did not receive any activities and went about their activities as usual	Yes	7 week teacher training program	7 weekly lessons on ability EI competencies, using either face-to-face approach or game-based e-learning approach
Pozo-Rico and Sandoval (2020)^4^	Teachers in a Primary School	Not measured	Yes	No, control group participants did not receive any activities and went about their activities as usual	Yes	14 week teacher training program	14 weekly lessons on various ability EI competencies

3 *This study was originally published in Spanish*.

4 *The authors did not mention where ability EI measure were used. The training methodology was based on the ability EI approach*.

### Approach 1: Teaching Ability EI

#### Educational Training Format

All eight MSCEIT studies included in Kotsou et al. (2019) meta-analysis consisted of a formal training workshop or a session dedicated to educate participants about a specific EI ability. The topics discussed within these sessions could be varied though supposedly grouped under a common theme (e.g., managing anger or self-management of negative emotions). For example, Clarke (2010a) EI training on “self-awareness” had 80 participants attend a morning and afternoon presentation on this topic. Participants had to do two individual exercises where they practiced recognizing microexpressions of emotions and emotional self-awareness. They also had to do two group exercises where they learned how to better understand emotions and how to recognize impulsive behaviors.

Similarly, Peter and Brinberg (2012) had 120 undergraduate students attend either an EI training workshop or a “food portion” workshop. Both workshops were equal in duration (75 min) or structure, except that the EI workshop had emotional content embedded into its teaching material. For example, the EI workshop has information about emotional eating (“why diets do not work”), while participants in the non-EI workshop attended sessions on food portions. While the authors did not provide an outline of the workshops' teaching content, their training was based on the principles set out by Cherniss and Adler (2000) or Elias and Arnold (2006). In their book on social–emotional learning, Elias and Arnold (2006) provided useful methods to teach these skill sets to students, such as the use of self-reflection, group activities, and informational sessions on key concepts. Peter and Brinberg (2012) adapted these principles and applied them to the four branches of the MSCEIT. As such, these training sessions generally followed the procedures found in Clarke (2010a) and previous ability EI-training studies.

Ability EI training could also be based on an existing social-emotional educational program. Castillo-Gualda et al. (2017) had 32 teachers from a private school in Madrid, Spain, completed 24 h of social–emotional learning (SEL) training across 3 months. They used an SEL program called the RULER approach (Brackett and Rivers, 2013), which is said to teach social–emotional skills based on the ability EI model by Mayer and Salovey (1997). The program itself consists of a series of activities, tools (e.g., worksheets), and educational materials around a specific emotional competency (e.g., mood management). When compared with teachers from a different private school in the area, the participants who completed the RULER training reported significantly higher EI scores on the MSCEIT Branch 1: Emotional Perception, Branch 3: Understanding Emotions, and Branch 4: Managing Emotions. The authors concluded that an SEL educational program had a positive impact in improving teachers' ability EI.

More recent training studies had been completed using a similar approach and with a substantially large sample of participants within and between countries. For example, Gilar-Corbí et al. (2018a); Gilar-Corbí et al. (2018b), Gilar-Corbí et al. (2019a) utilized a training protocol called the “Emotional Intelligence Training Program.” In these studies, training activities consisted of 60–95 min of weekly sessions over 6 or 7 weeks. The first session is always to provide an introduction of EI. Subsequent sessions focused on teaching and practicing competencies related to the ability EI model. For example, in Gilar-Corbí et al. (2018a), Session 2 consisted of training in emotional perception and the sixth and seventh sessions were on stress management and emotional management, respectively. While each of these training studies differed slightly with respect to the sample group and session length, they all reported improvements between the pre-training and post-training tests of ability EI, such as on the STEU or STEM (Gilar-Corbí et al., 2018a) as well as on the MSCEIT (Gilar-Corbí et al., 2018b).

When reviewing the list of ability EI training studies with those obtained from Kotsou et al. (2019) study, it was clear that almost all of these studies involved participants completing several informative sessions. These sessions tend to be educational in content and format type (e.g., lectures, workshops), with a trained instructor providing information regarding specific EI abilities. In Gilar-Corbí et al.' set of studies, a researcher trained in their version of social–emotional program provided the training. A predetermined amount of time would then be set aside for participants to practice these abilities (e.g., using role-plays) or to self-reflect on this information. In their meta-analytic review, Hodzic et al. (2018) identified these sessions as theory-based (e.g., using lectures or case studies to teach about the theories of EI) and the activities as experience-based skills practice. Given the format, these EI training sessions typically required massed participation by a group of people engaging in the learning activities at the same time. Subsequent practice activities were then used to reinforce the concepts taught in the theory-based sessions.

#### Constellation of Training Activities

A related theme for these ability EI training studies was the use of multiple and very different activities during the intervention period. For instance, Codier et al. (2011) used a different teaching paradigm (specifically peer coaching) instead of a more traditional workshop format. They suggested that peer coaching, in which two professionals converse and reflect, to be more effective in improving the EI of the participants. In their study, nurse managers working in a medical center were asked to select a professional peer and to pair with them over a 6 month period. They were told to identify one or two emotional skills to work on. These dyad groups met for weekly peer coaching sessions where they listened and reflected on topics and experiences relating to these skills. Additionally, monthly informational meetings about related emotional skills were also conducted. Despite the difference in format, these meetings were similar to those used in other EI training workshops. That is, a specific emotional skill was openly highlighted, discussed, and practiced using appropriate exercises or informational sessions.

Meyer et al. (2004) also provided a different take on the traditional workshop format by using an experiential training program. They had executive-level employees of a mid-size dental practice complete a “novel and challenging” adventure program that involved indoor physical activities (e.g., ropes and obstacle course) that required team cooperation for problem solving. Importantly, Meyer et al. had facilitators to provide discussions regarding the experiences of the participants, to develop the meaning behind these activities, and then to extend this knowledge to the workplace and personal life. The skills involved in this intervention were around interpersonal communication (e.g., recognizing verbal and non-verbal cues) and motivational goals (e.g., working toward fulfilling a common task). Despite the slight difference in training format, Meyer et al.'s adventure training was reported to have improved the emotional competencies of the participants as measured using the MSCEIT before and after the intervention. However, the small sample size (*n* = 15) and the non-significant findings after Bonferroni adjustments made it difficult to attribute these improvements to the training program.

Similarly, the EI training utilized by Gilar-Corbí et al. (2018a); Gilar-Corbí et al. (2018b), Gilar-Corbí et al. (2019a) added on the traditional educational format. Apart from completing specific EI-related exercises based on the targeted domain for that session, participants can either complete further activities on an electronic-learning platform or receive personalized and individualized coaching. Overall, it would appear that participants who completed the specific EI training plus a second modality (e.g., personalized coaching) reported the most gains in ability EI (Gilar-Corbí et al., 2018b). While Gilar-Corbí et al.'s studies demonstrated that ability EI could be trained in this manner, the lack of matched active control groups hampered the utility of these findings. Although the participants in the control groups were blind to the training conditions and continued about their usual activates, these activities were not matched to the ability EI training activities. For example, control group participants in Gilar-Corbí et al. (2019b) study completed the same pre- and post-measures as the training group, but were not given access to the EI training.

A recent study by Geßler et al. (2020) provided contrary evidence to EI training using this broad approach. They attempted to address several of the key weaknesses by having an active control group and a 4 month follow-up assessment after training completion. Geßler et al. had three groups of participants, with groups 1 and 2 each completing a different type of emotion regulation training, and Group 3 being the active control group. Each group completed 1 day of workshop based on the training condition assigned and thereafter 4 weeks of online group sessions. The group sessions were small (<10 people) and involved teaching, activities, and sharing. Group 1 completed training activities specifically designed to improve abilities to perceive and regulate emotions in self and others. The activities included information about emotion regulation strategies and the self-regulation of positive and negative emotions. Group 2 completed the same training activities, with the exception of having two additional practice exercises to do each day. Group 3 completed activities that focused on presentation skills or time management skills. The duration allocated to practicing these skills was similar to groups 1 and 2. Using multiple-group Structural Equation Modeling analyses, Geßler et al. found no significant changes in the MSCEIT scores of the participants across all training conditions. This pattern of results persisted after a follow-up assessment (i.e., MSCEIT data at the end of the intervention and 4 months later were compared).

#### Length of Training Completion

One other common feature of these training programs is the long duration of the program; because of the theory-based and experience-based features of these training studies, they often required many months or even years to complete. Dacre Pool and Qualter (2012) had undergraduate students complete 11 weekly classes of ability EI activities, with each session lasting 2 h. Many different types of activities occurred in these classes, including mini-lectures, case studies, group work, videos, role-plays, discussions, and a visit to an art gallery over 3 months. The authors provided an example of training Branch 4: Managing Emotion on the MSCEIT. First, a lecture on emotion management was conducted to provide information regarding this topic (e.g., why is it different from suppressing one's emotion?). Students were given an article to read on their own, which contained research findings related to anger management. Group work was then conducted to discuss case studies and personal experiences of poor emotional management. This was followed by role-playing exercises for students to practice the use of effective strategies and what they had learned from the above-mentioned activities.

A similar training protocol was found in Martyniak and Pellitteri (2020) study, in which they conducted EI training for Preschool Teachers in Poland. These teachers completed 3 full-day EI training workshops over 3 months. They were also provided with short daily exercises to train upon each week (5 times). These exercises were said to target each branch of ability EI. While the results demonstrated improvements in MSCEIT scores post-training, it was uncertain how these improvements occurred. The training interventions consisted of two distinct components: (a) The lectures on EI-related abilities and (b) the follow-up activities (daily or weekly). This format raised several questions: (1) Were the lectures necessary to impart knowledge; (2) were the follow-up activities sufficient for improving ability EI, as suggested by Martyniak and Pellitteri (2020); and (3) if so, to what extent were the improvements attributed to either training components.

The study duration of the EI training using this approach can also take years to fully complete. Ruiz-Aranda et al. (2012a,b) reported the improvements in the psychosocial outcomes, psychological adjustment, and mental health functioning in adolescents after completing the INTEMO Project (Ruiz-Aranda et al., 2008)^2^. According to the authors, the INTEMO training was administered to students (12–17 years of age) studying in the Spanish Compulsory Education System over a time period of 2 years. The actual EI program itself took place between January and June in 2009 and 2010, with the post-treatment data obtained in June 2010 and a follow-up assessment in December 2010. It involved 24 hourly sessions scheduled during a regular school day as well as a 3 h training for each branch of EI as defined by MSCEIT. Training activities were multi-modal and were designed to help students learn ability EI through the use of role-playing, art, film discussions, and personal reflection. An example of Branch 4: Managing Emotion training could be “learning adaptive ways to handle emotions and creating a forum to discuss strategies” (Ruiz-Aranda et al., 2012a, p. 464). Ruiz-Aranda et al. reported that students who completed the EI training demonstrated improvements on a wide variety of outcomes, such as perceived stress, satisfaction with life, and subjective happiness. The results indicated that these training programs could be used to develop the skills associated with ability EI. However, the authors did not include measurements of ability EI or trait EI to chart the changes in EI-related competencies. Thus, it was not possible to conclude whether improvements in ability EI were the mediating factors in changing these life outcomes.

#### Review of Approach 1

Regardless of its efficacy, the commonality across these EI training studies seemed to be on the development of long-term emotional knowledge through instruction and educational learning. For example, participants were specifically instructed and given opportunities to practice the effective strategies to manage their own or other people's emotions (Dacre Pool and Qualter, 2012). These strategies should correspond to Branch 4: Managing Emotions on the MSCEIT. Since ability EI is supported by emotional knowledge as well as the associated abilities relating to this knowledge (Mayer et al., 2016), improvements using this training approach possibly occurred because of an increase in the long-term memory for emotional information. Evidence for this approach could be observed in cross-sectional studies that demonstrated a strong relationship between ability EI and crystallized intelligence (Gc), as well as minor-to-moderate relationship with fluid intelligence (Gf) (MacCann, 2010; Olderbak et al., 2019).

Several notable weaknesses and limitations constrained this method of improving EI, although these limitations were perhaps inherent within the protocols. For example, it would be challenging to have active control groups in training paradigms that utilized workshop-style intervention protocols. Many of these training studies often utilized professional participants (managers, professional athletics, or teachers). Second, these workshops tend to use very diverse learning materials and teaching modalities, such as feedback, role-plays, presentations, practice sessions, and even outdoor visits and physical training (e.g., visiting an art gallery; Dacre Pool and Qualter, 2012). The costs and logistical demands of conducting such training interventions would be considerably high and time-consuming. Finally, these training features also made it very challenging to identify the mechanisms and processes underlying these improvements. For example, there was no proper theoretical account as to why Meyer et al. (2004) adventure training would improve ability EI apart from the emphasis on how experiential training could improve social–emotional competencies.

### Approach 2: Brain-Training EI

#### Computerized Brain Training

The earliest example of EI training using this approach was by Eack et al. (2007). Between August 2001 and January 2006, they recruited participants from several outpatient and inpatient schizophrenia services. These participants either were at the early course of schizophrenia or diagnosed with schizoaffective disorder. Participants completed sessions based on either the Cognitive Enhancement Therapy (CET) or Enriched Supportive Therapy (EST) (see Hogarty et al., 2006 for detailed descriptions of both therapy approaches). Importantly, participants in the CET group completed ~60 h of computer training in attention, memory, and problem solving. When compared to the participants in the EST group, they demonstrated large improvements in overall EI. They also demonstrated significantly higher scores on the MSCEIT Branch 2: Using Emotions; Branch 3: Understanding Emotions, and Branch 4: Managing Emotions. There were no significant differences between these two treatment groups in terms of age, gender, and baseline IQ. Eack et al. (2007) concluded that CET was able to improve the participants' abilities to understand and manage emotions in themselves and others. However, a key limitation of this study was that participants in the CET group also completed 45 weekly group therapy sessions. These group sessions included a number of different activities that focused on teaching participants about taking the perspective of others, reading non-verbal cues, and managing or accurately appraising their own emotions or others. As such, it is unclear whether the improvements on the MSCEIT scores were due to the computerized training, lessons on emotional abilities, or a confluence of both.

To partially address this issue, Hooker et al. (2013) utilized a combined computer-based training invention that targeted social cognitive abilities. They had similar participants complete ~60 min of Auditory Training (AT) and Social Cognitive Training (SCT). The AT program consisted of a series of computerized exercises to improve auditory and verbal information processing. The SCT program consisted of several computerized exercises taken from two commercial training packages, all of which were designed to engage participants' perceptual and executive control processes related to emotion recognition. A separate control group of participants completed 60 min of daily computer game (CG) placebo. Sixteen commercially available games (e.g., solitaire, checkers, hangman) were rotated according to a schedule given by the researchers. The results indicated that participants that completed the AT+SCT training had significant improvements on the MSCEIT Branch 1: Perceiving Emotions. This improvement was also associated with increased activity in their left amygdala, putamen, and the medial prefrontal cortex. The participants in the control group, who had only trained on the video games, did not demonstrate any of these improvements. Hooker et al. concluded that such brain training interventions improved participants' theory of mind by intervening on the neurocognitive systems for emotional recognition.

A follow-up study by Nahum et al. (2014) used an online training program called SocialVille (Brain Plasticity Institute of Posit Science; Nahum et al., 2013). The training itself consisted of 19 computerized exercises that targeted speed and accuracy in processing social information. According to Nahum et al. (2013), these exercises specifically intervened at the cognitive domains of emotional perception (visually and verbally, social cues appraisals, Theory of Mind, and self-reference). Each training session had 6 exercise blocks and each block takes about 10 min to complete. The total training duration was 24 h or ~1–2 h per day for 6–12 weeks. Interestingly, Nahum et al. (2014) found that participants that completed the SocialVille program did not significantly improve on Branch 1: Perceiving Emotions and Branch 2: Managing Emotions on the MSCEIT. The authors concluded that the focus of the training exercises was on processing speed, rather than on accuracy only. The MSCEIT is not a timed task; as such, it might not have fully captured participants' improvements in emotional perception. Additionally, the duration of the SocialVille training (i.e., 10 min each day) was substantially much shorter than previous cognitive training studies. Nevertheless, these participants demonstrated improvements on the SocialVille tasks measuring emotional recognition and perception. Nahum et al. (2014) further suggested a need for future cognitive training on EI to be specific about what the underlying processes being trained as well as to increase the daily training dosage.

#### Review of Approach 2

Approach 2 used in EI training studies focused on directly improving the neuro-cognitive processes underlying EI. The training tasks utilized in these training studies also tend to be singular in format (e.g., the use of computerized programs) as compared to the other training studies that utilized a large array of activities (e.g., role-plays, workshops, video watching, lectures, reflections). It should also be apparent that EI training studies using this approach had been predominantly conducted using diverse population groups. As such, they were dissimilar to many existing studies on EI training. None of these studies appeared in Kotsou et al. (2019) and Hodzic et al. (2018) meta-analytic reviews.

## Discussion

The current narrative review sought to identify the features and broad approaches used in ability EI training studies. Three features were present in almost all of the ability EI training studies conducted with healthy adults and adolescents. We classified these features as Approach 1: Teaching ability EI. The commonality across these studies were that they utilized an educational instruction format as well as using a constellation of different activities during the training period. We classified a fourth feature as Approach 2: Brain-training EI. This feature was only found in training studies that explicitly utilized cognitive training principles. The findings obtained in the present review demonstrate that ability EI can be trained and improved upon. More importantly, these studies provide several insights into the theoretical and practical implications of EI training for future EI researchers. In the next section, we explore these implications from these two approaches.

## Theoretical Implications

Ever since Goleman (1995) popularized the concept of EI as being as important as cognitive intelligence, scientific interest in EI has exploded ever since. A large number of studies have documented the relationship between EI and various life outcomes. Training studies, in particular, provided invaluable insights into the theoretical components of ability EI. Performance on ability EI tests can be attributed to the assessment methods used (MacCann and Roberts, 2008) and not of the construct itself. Ability EI is usually measured using maximum performance tests (similar to other intelligence tests). However, unlike traditional IQ tests, one of the biggest challenges is on determining how to score a correct response on an ability EI test such as the MSCEIT. EI is largely an individual difference construct, and scientific examination had often been restricted to correlational approaches. This, plus other challenges, further limits the criterion validity of EI (Fiori and Vesely Maillefer, 2018). Training studies, however, provide a complementary methodology to study the contributions of ability EI using an experimental approach. This allows us to specifically examine training-induced changes in performance (e.g., emotional perception), which then gives us reasons to conclude that emotional perception is a core component of ability EI.

### Teaching Ability EI: Multimodal Methods

Because of the varied activities and modalities used in the training studies classified in this approach, it is hard to determine which processes are being targeted in the EI training. At the very least, many of these studies appeared to be targeting participants' long-term emotional knowledge and competencies, with educational activities being a large part of the training protocol (Peter and Brinberg, 2012; Gilar-Corbí et al., 2018a). Gilar-Corbí et al. (2019a) suggested that EI training using this approach needed increased time and more exercises to produce larger training effects. They also suggested that participants needed more experience and opportunities to practice these skills. These aspects of instructional and educational format were all mirrored in these training studies. The general purpose is to “teach skills”; but unlike teaching technical or academic skills, the central goal of these studies is to impart emotional competencies that would be beneficial in overcoming obstacles in life (Gilar-Corbí et al., 2018a).

Importantly, these studies demonstrated that an approach aimed at improving long-term knowledge for emotional competencies could be effective in improving some aspects of ability EI. Particularly, several of these studies extend the existing literature of ability EI training by providing useful suggestions on methodical improvements. Instead of utilizing only multiple training activities, several recent training studies incorporate multi-modal delivery. For example, Gilar-Corbí et al. (2019a) utilized both face-to-face training and a virtual support system among participants (i.e., senior managers). Likewise, Gilar-Corbí et al. (2018a) incorporated an online-exclusive modality condition and a coaching condition, in which individualized training could be provided to each participant. Similarly, a multimodal style could also be found in Martyniak and Pellitteri (2020) study.

Importantly, a multimodal approach may also address several of the notable limitations. The costs and logistical demands of conducting such training interventions could be high. Recent training studies using different modalities (online or face to face) indicated that each of these modalities could be effective in improving ability EI. If so, EI training can be tweaked to provide a certain degree of flexibility and according to the specific needs, resources, and opportunities available to that particular population group. For example, students can incorporate online EI learning as part of their normal curriculum, without detrimental consequences to their overall learning (Gilar-Corbí et al., 2018a).

### Brain Training Approach to Ability EI

We were surprised to find that none of the training studies in this approach were identified in either Kotsou et al. (2019) or Hodzic et al. (2018) meta-analytic reviews. In fact, it seems that there have been no published studies on improving ability EI utilizing a cognitive or brain training approach and with a non-clinical sample. This is in contrast to the wider literature on cognitive training, where a number of studies have examined training effects on fluid reasoning and executive functioning (Jaeggi et al., 2009; Webb et al., 2018). While training ability EI using this approach is at its infancy, we argue that this approach provides several advantages. Recent experimental and cross-sectional studies had demonstrated that such EI abilities were implicated in basic cognitive tasks (Gutiérrez-Cobo et al., 2017a; Lim and Birney, 2021). Additionally, a moderate relationship has been found between ability EI (but not trait EI) and performance in cognitive tasks that involved emotional information, such as the Iowa Gambling tasks (Checa and Fernández-Berrocal, 2019), emotional go/no-go task (Gutiérrez-Cobo et al., 2017a), and emotional face N-back task (Gutiérrez-Cobo et al., 2017b). Megías et al. (2017) also found that participants with higher EI displayed a larger N2 component on Event-related Potential (ERP) readings during the emotional go/no-go task, suggesting a greater capacity for cognitive control, detection, and evaluation of emotional stimuli.

Lim and Birney (2021) found that ability EI contributed to participants' ability to inhibit flanking distractors. At the same time, participants' inability to suppress emotional distraction seemed to correspond to their performance on the MSCEIT, particularly on Branch 4: Managing Emotions (Gutiérrez-Cobo et al., 2017b). These findings raise an important question of whether training these basic cognitive processes (e.g., cognitive control) could positively affect one's ability EI or vice versa (Megías et al., 2017). At the same time, using the cognitive training approach allows for a finer-grained examination of any observed improvement in EI ability by using theories from the cognitive training research, such as (1) Near or far transfer; (2) plasticity; and (3) process-specific or skill-based improvements (Gathercole et al., 2019).

#### Near and Far Transfer

A cornerstone in the cognitive training literature is the concept of “near” and “far” transfer effects (Katz et al., 2018). Briefly speaking, near transfer refers to the improvements attained through the processes that are being directly trained by the person. On the other hand, a far transfer is attained when improvements other than the targeted area are observed. In reviewing all the past studies on EI training, many did not specifically discuss whether the interventions had brought about near or far transfer effects. The use of transfer-related terms can help to express the usefulness of the interventions being used. For example, in addition to improving a person's attention and cognitive control (a near transfer), meditation training could also improve a person's learning and retrieval of information (a far transfer) (Mrazek et al., 2013).

Near and far transfer effects in the training literature are not only used to determine the usefulness of the intervention itself. Rather, these transfer effects provide important information concerning the theoretical bases regarding the underlying processes that drive these improvements and of the construct itself (Sala et al., 2018). If EI training improves performance on a task measuring emotional inhibition, this finding contributes to the theoretical conceptualization of EI as a set of cognitive abilities that are used for emotional control. Many of the previous ability EI-training studies did not include different measures of emotional competencies other than the MSCEIT itself. This reliance had been a key limitation concerning the theoretical aspects of these interventions as well as for the broader EI construct. A cognitive training approach to EI improvements can help to address this limitation. For example, EI training researchers can utilize different cognitive tasks that examine how participants process and manage emotional information (e.g., emotional go/no-go task, Megías et al., 2017; emotional blink task, McHugo et al., 2013; emotional flanker N-back task, Lim and Birney 2021). Together with pre- and post-training data using the MSCEIT, STEU, or STEM, the results can provide information regarding the mediating effects of the specific training and the specific emotional processes being targeted.

#### Neuroplasticity

One of the more appealing theories in the cognitive training approach is that the observed EI-related gains after training reflect the plasticity in the cognitive-neural system underpinning EI. The evidence for this came from past neuroimaging studies of EI (Krueger et al., 2009; Barbey et al., 2014). Interestingly, Krueger et al. (2009) found that damage to the ventromedial Prefrontal Cortex (PFC) reduced the Strategic EI score on the MSCEIT (i.e., combined from Branch 3 and Branch 4). Additionally, damage to the dorsolateral PFC reduced the Experiential EI score (i.e., combined from Branch 1 and 2 on the MSCEIT). In another study, brain activations were also much reduced when individuals with higher EI had to process and manage emotional information when working on the Wason card task (Reis et al., 2007). These imaging studies suggested a unique neuro-cognitive structure for EI and one that could be complementary to cognitive intelligence. If so, training that specifically targets these cognitive processes within this structure will likely yield improvements in ability EI. Evidently, this line of reasoning had been taken up by the three cognitive training studies reviewed in this article (Eack et al., 2007, Hooker et al., 2013; and Nahum et al., 2014).

At the same time, the theory of neuroplasticity may also account for the improvements in ability EI following an increase in the long-term emotional knowledge (Approach 1). Long-term knowledge and emotion-related abilities had been found to be implicated in various areas of the brain (Dalgleish, 2004). Knowledge regarding emotional concepts, moods, and social expressions were typically provided and practiced over multiple sessions in this training approach. If the neural structure of emotional abilities improved with knowledge and practice, its benefits should also extend to improvements in EI-related outcomes. In fact, if we were to include the EI-training studies that did not use the MSCEIT in our review, the evidence base for EI-training substantially increased. Kotsou et al. (2019) and Hodzic et al. (2018) data indicated that EI could be improved through training and that one possible reason is that the neuro-cognitive system supporting EI is malleable to a considerable degree.

#### Process-Specific Changes or Skill Acquisition?

A related theory regarding EI training effects is that it enhances specific processes within EI abilities. For example, emotional perception is a key competency measured by the MSCEIT. Cognitive training using perceptual tasks may be useful to improve the efficiency and accuracy of cues relating to emotional information (Nahum et al., 2014). Another interesting approach is the training of executive functioning processes. Three core executive functions are frequently identified in the wider executive function literature, namely: (1) Inhibition; (2) Updating; and (3) Cognitive Flexibility (i.e., the unitary model; Miyake et al., 2000). Ability EI had already been associated with performance on several executive functioning tasks, with higher EI scores positively associated with performance in inhibition tasks and updating in adults (Lim and Birney, 2021). Such findings raise several important questions: (1) whether EI abilities such as understanding emotions or managing emotions can be trained using cognitive inhibition tasks or updating tasks; and (2) whether we can modify existing cognitive training protocols to improve ability EI. The findings by Gutiérrez-Cobo et al. (2017) or Lim and Birney (2021), which both linked ability EI with working memory, were intriguing because the implication was that training the latter might also affect the former. For example, process-specific changes within emotional-related abilities could lead to an increased efficiency in which decisions about emotional states were made (Schweizer et al., 2011). Dahlin et al. (2008) suggested that effective transfer occurred when the criterion and transfer tasks overlapped in processing components and the corresponding brain region. If so, training that targeted the specific parts of the brain for emotional information (e.g., face perception) could be used to improve one's ability EI, specifically on Branch 1: Perceiving Emotions.

The cognitive training literature also suggested that skills acquisition through instruction and practice could also be the primary mechanisms in which improvements occurred (Willis and Schaie, 2009). One of the most common examples was the use of appropriate strategies. A strategy can be defined simply as the alternative method for performing a particular task. For example, many past memory-training studies specifically instructed the method of loci as a means to improve the participants' recall (Yesavage, 1990). Likewise, the use of visualization, imagery, or organizational principles could be described as useful skills or strategies that could be used to train participants' memory (Willis and Schaie, 2009). In the cognitive training literature, the use of cognitive control strategies to improve working memory had also been found to improve emotional working memory (Schweizer et al., 2011). In which case, similar strategies may be helpful in facilitating useful responses when dealing with emotional situations. This would be consistent with abilities measured by Branch 2: Using Emotions on the MSCEIT (Mayer and Salovey, 1997).

### Practical Implications: What Future EI Studies Need to Do

A crucial issue for future EI training research is how to conduct training studies that address the following two questions that motivated this review:

- What processes and abilities are targeted in EI training interventions?
- Whether there is a method to improve EI abilities using a cost-effective or economical way while still retaining the rigor, reliability, and validity expected of psychological research?

While the answer to the above two questions is perhaps non-exhaustive, we proposed two key areas that must be addressed. The first is the inclusion of active control groups in all future EI training studies. The second is that the durability and generalizability of EI improvements should be examined as well.

#### Active Control Groups

Geßler et al. (2020) correctly pointed out that past EI training studies were lacking in experimental rigor, active control groups, and appropriate comparisons. These limitations were also observed in Kotsou et al. (2019) list of studies used for their meta-analytic review, where many lacked an active control group and randomization of participants. Other more recent studies had used control groups, but many of these participants went through their usual activities as per normal (Gilar-Corbí et al., 2018a, 2019a). In other words, many of these EI training studies could only provide pre- and post-training results within the training groups without any other comparisons with appropriate control groups. This made the interpretations of any training effects on EI extremely challenging, as these improvements could have been incidental due to placebo or Hawthorne effects. These effects had already been found to severely confound past studies on cognitive training (Shipstead et al., 2012). For example, Geßler et al. (2020) training study failed to produce any improvements in MSCEIT scores after including active control groups and appropriate follow-up assessments.

To combat this issue, it is important for future EI-training studies to include an appropriate control group to compare the efficacy of the EI interventions. We proposed that a cognitive training approach to EI training can address this limitation. For example, it is considerably easier to have a separate control group of participants complete non-EI training activities (e.g., using computer games, Hooker et al., 2013). This can be inexpensive to implement, as these games are often commercially available or easily accessible to participants. Additionally, an active control group can be trained using the existing cognitive training paradigm (e.g., dual N-back training; Jaeggi et al., 2009). Another example is that researchers can compare whether emotional N-back training is effective in improving ability EI as compared to a non-emotional N-back training. The addition of active control groups allows for a more scientifically rigorous methodology to investigate EI training effects. It also allows for true randomization of participants in a training program, as compared to simply randomly choosing a cohort (e.g., class) into training and control conditions.

#### Durability and Generalizability of Training Effects

A final consideration is regarding the durability of EI-training effects. Without clear guidelines on control groups and transfer effects, it is impossible to determine the durability of EI-training effects in the mid- to long-term after the completion of training. As mentioned earlier, many of these training studies either lacked an appropriate follow-up component (Codier et al., 2011, 2013) or measured participants using the same EI measures post-training (Nelis et al., 2009, 2011). Again, we proposed that future EI-training studies that utilize the cognitive training paradigm could incorporate cognitive measures that tapped into other cognitive–emotive processes. For instance, ability EI was found to be a significant predictor for participants' N-back updating for emotional faces, but not for neutral stimuli (Gutiérrez-Cobo et al., 2017b). Alternatively, researchers could use a different variant of the WM task (e.g., using emotional words as probes) to better determine the generalizability of EI-training effects. These will shed much light on the cognitive processes being trained and whether these improvements persist beyond the immediate end of the training.

## Limitations

The present review has several limitations that require mention. First and due to the nature of narrative reviews, we did not include any data on the efficacy of EI training studies identified in either of the two approaches. Since Kotsou et al. (2019) and Hodzic et al. (2018) meta-analytic reviews, a number of recent studies — notably by Gilar-Corbi and colleagues as well as Pozo-Rico and Sandoval (2020) had been published. While these studies would still be identified as having similar features in Approach 1: Teaching Ability EI, they incorporate multi-modal elements in the training delivery. These researchers reported modest gains in ability EI. As such, it would be helpful to examine the efficacy of these newer training studies. It also suggested that refinements to teaching ability EI can address the inherent limitations noted in this review.

A second limitation is that we did not actively identify the tasks used to determine training gains other than tasks that measure ability EI (e.g., MSCEIT, STEU, or STEM). In other words, we did not wholly consider that improvements in EI training could have on other variables. Training ability EI cannot simply be an exercise by itself, and that EI gains do in fact lead to improvements in other variables such as academic functioning or job satisfaction. We also did not actively consider the specific population groups that participants come from (e.g., students or business managers), partly because we considered them as possible moderator variables to be used in a meta-analytic review. Future systemic or meta-analytic reviews of this topic can consider using these moderators and different outcome measures to determine the effectiveness of ability EI training.

## Conclusion

We ask ourselves a fundamental question: Can ability EI be trained? The answer is likely a yes and the evidence for this has already been well-documented by previous meta-analytic studies. However, perhaps a different question should be asked instead: What makes the training of ability EI possible? In our current review, we identified commonalities or prototypical features found in previous ability EI training studies. After classifying these features, there appeared to be two broad approaches utilized in the training of ability EI. While both approaches may be equally effective in improving ability EI, we believe that a brain-training approach holds several advantages over the former. Nevertheless, we acknowledge that a teaching format in improving ability EI can be adapted and further refined. Using principles found in these two training approaches will open up new and exciting ways to conceptualize ability EI as well as to find novel methods to improve one of the key aspects of human functioning.

## Author Contributions

MLi conceived the presented idea and led this narrative review. MLa developed the theories and suggested the search and review strategies. All authors contributed to the design and implementation of the research, to the thematic extractions of previous studies, discussion of the results, and to the writing of the manuscript.

## Conflict of Interest

The authors declare that the research was conducted in the absence of any commercial or financial relationships that could be construed as a potential conflict of interest.

## Publisher's Note

All claims expressed in this article are solely those of the authors and do not necessarily represent those of their affiliated organizations, or those of the publisher, the editors and the reviewers. Any product that may be evaluated in this article, or claim that may be made by its manufacturer, is not guaranteed or endorsed by the publisher.

## Footnotes

^1^While several of the other ability EI training studies utilized computerized or web-based training, they used a multitude of other training activities concurrently or sequentially. As such, the use of computerized training was not a hallmark of these studies.

^2^The article by Ruiz-Aranda et al. (2008) was originally written in Spanish and not in the English language. As such, it was not included in this review.
